# Supplementary material for: Urotensin II Enhances Advanced Aortic Atherosclerosis Formation and Delays Plaque Regression in Hyperlipidemic Rabbits
Source: Int J Mol Sci. 2023 Feb 14;24(4):3819. doi: 10.3390/ijms24043819 (PMC9963243; doi:10.3390/ijms24043819)
Supplement: Supplementary file 1 [file ijms-24-03819-s001.zip › ijms-2201565-supplementary.pdf]

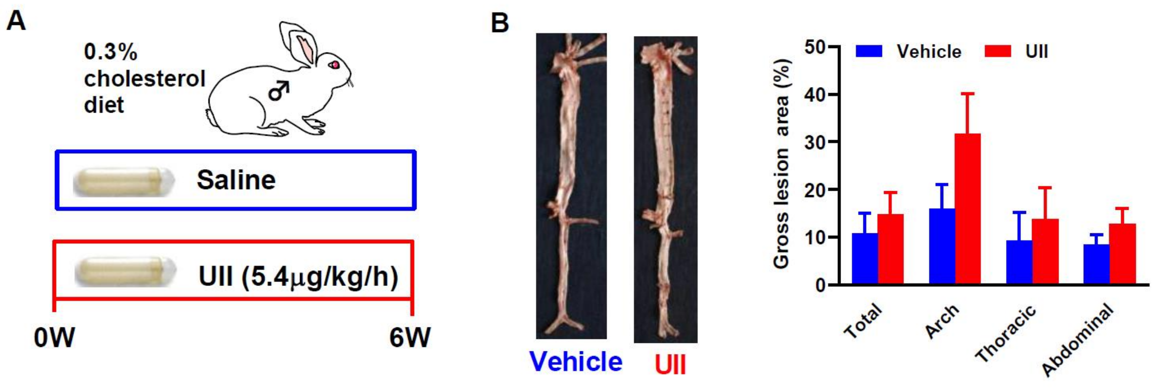

Figure S1. The effect of UII Infusion on atherosclerotic fatty streak in male rabbits. (A) Schematic illustration of the short-term male rabbits experiment. (B) The representative images of Sudan IV stained aortas and quantification of the sudanophilic area of the whole aortas. N=6 for each group, all data are presented as mean ± SEM.

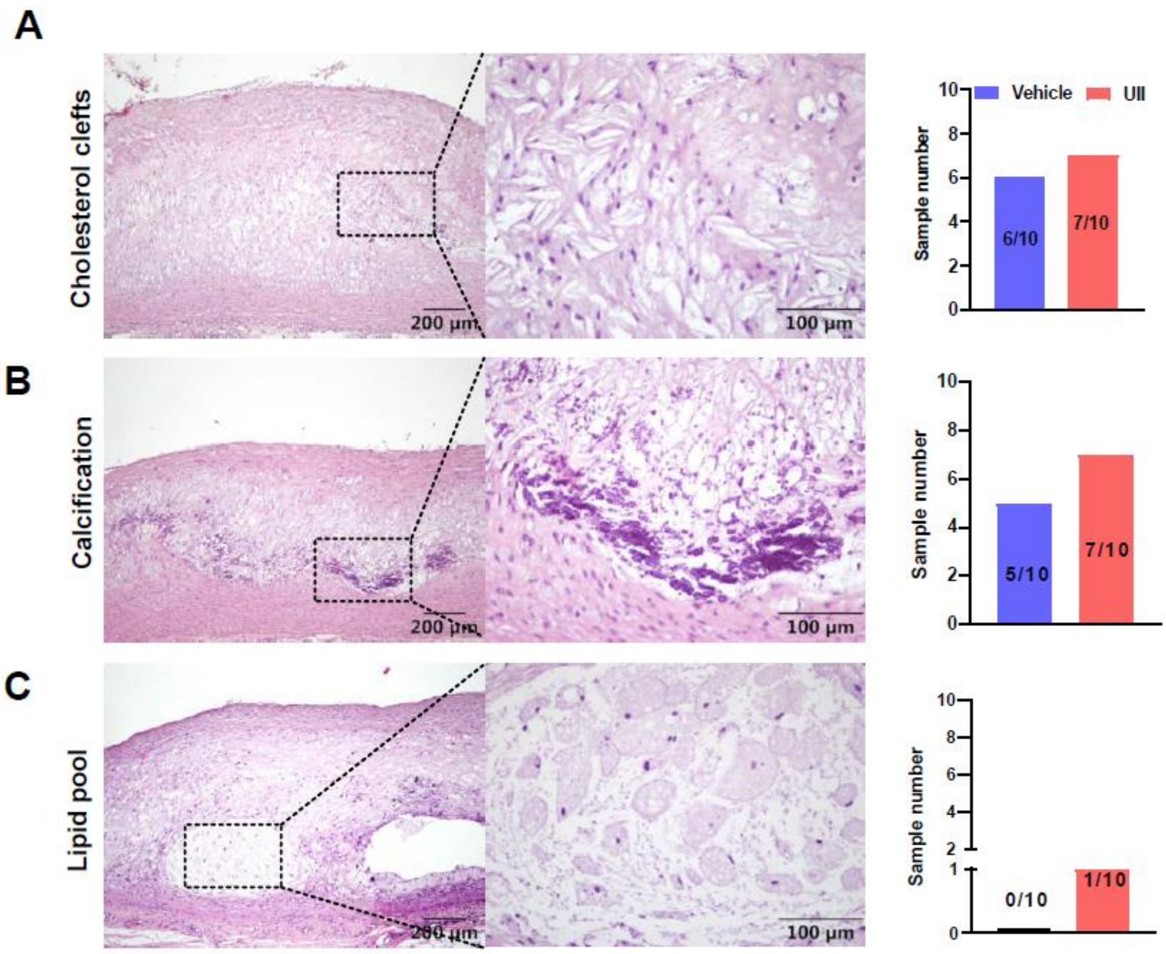

Figure S2. Histological characteristics of advanced aortic plaque in rabbits. (A) The representative images of intra-plaque cholesterol clefts. Number of rabbits with intra-plaque cholesterol clefts was calculated in both two groups. (B) The representative images of intra-plaque calcification. Number of rabbits with atherosclerotic calcification was counted. (C) The representative images of intra-plaque lipid core. Typical intra-plaque lipid pool was found in one UII infused rabbit.

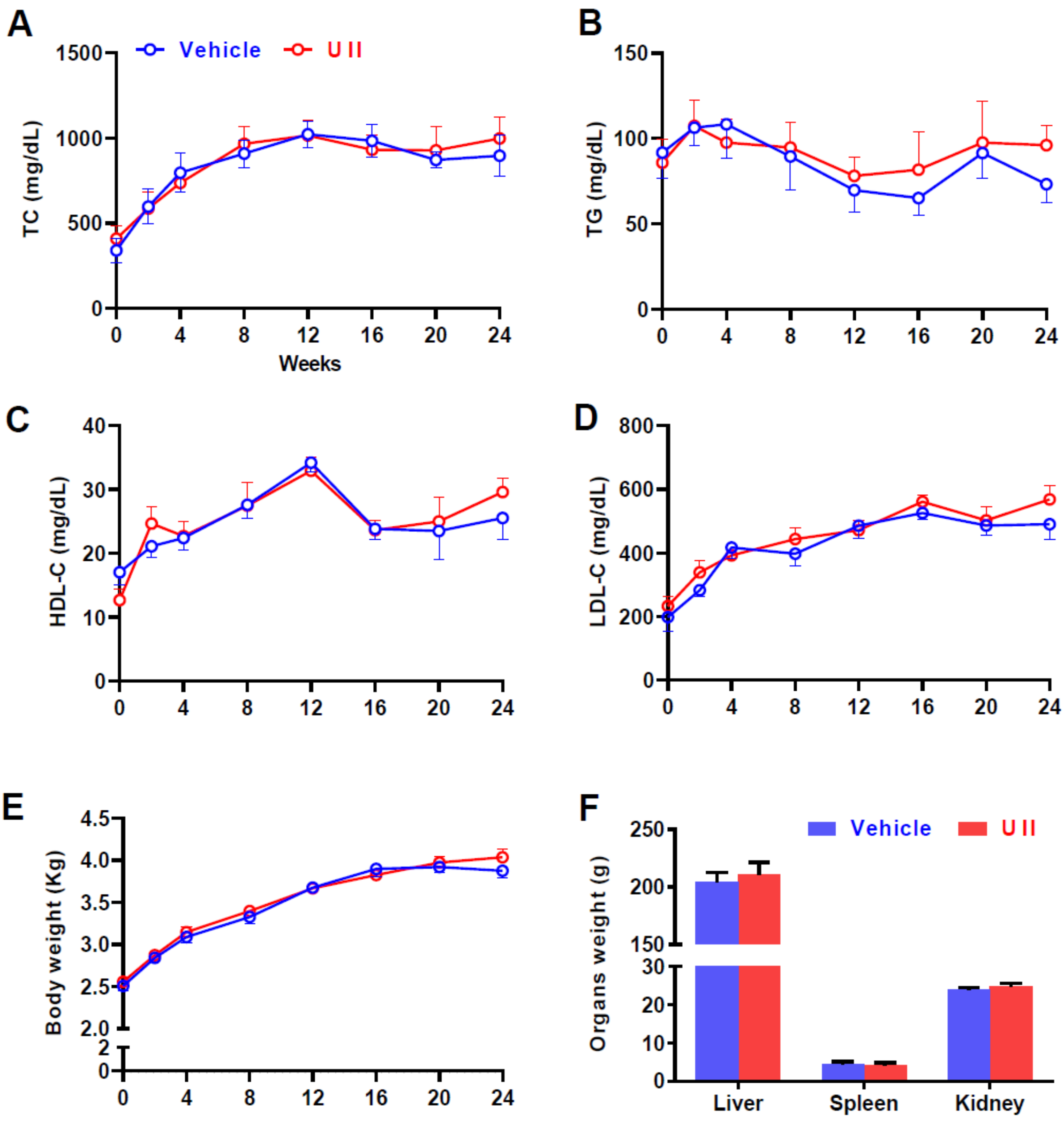

Figure S3. Plasma lipid levels, body weight, and organ weight of rabbits for advanced plaque study. (A-D) Plasma total cholesterol, triglycerides, high-density lipoprotein-cholesterol and low-density lipoprotein-cholesterol. (E) Changes in body weight. (F) Organs weights of HCD-fed rabbits. n=10/group. *p<0.05 vs vehicle group.

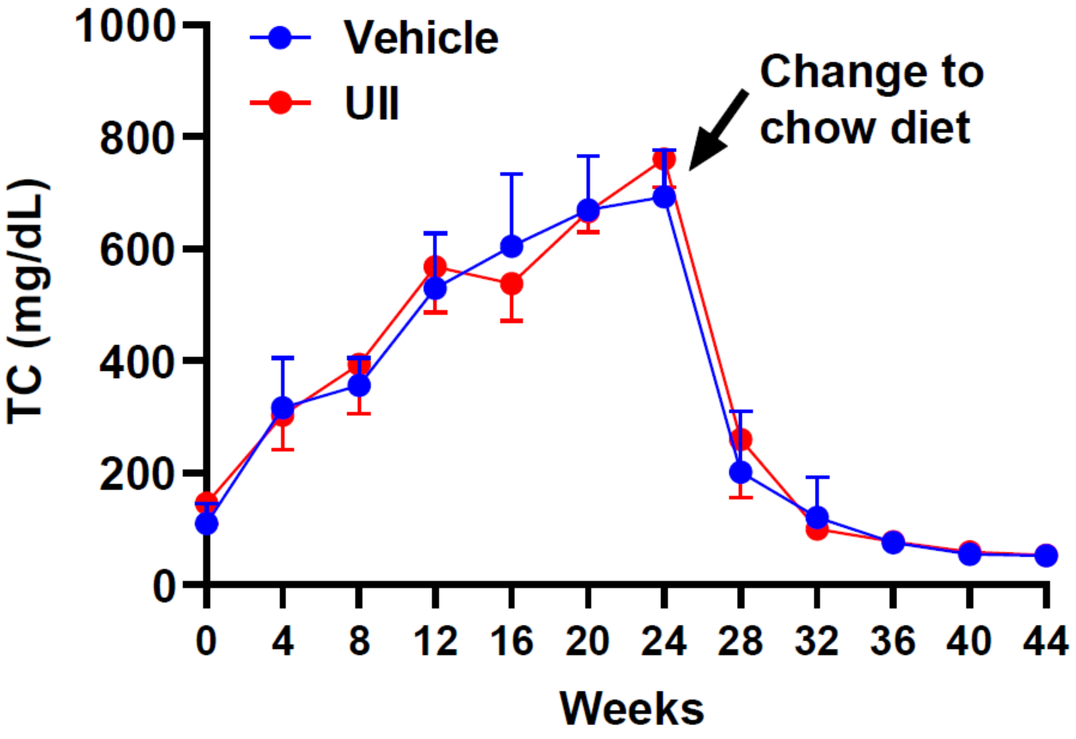

Figure S4. Plasma lipid levels of rabbits for plaque regression study. N=5-6 for each group. *p<0.05 vs vehicle group.

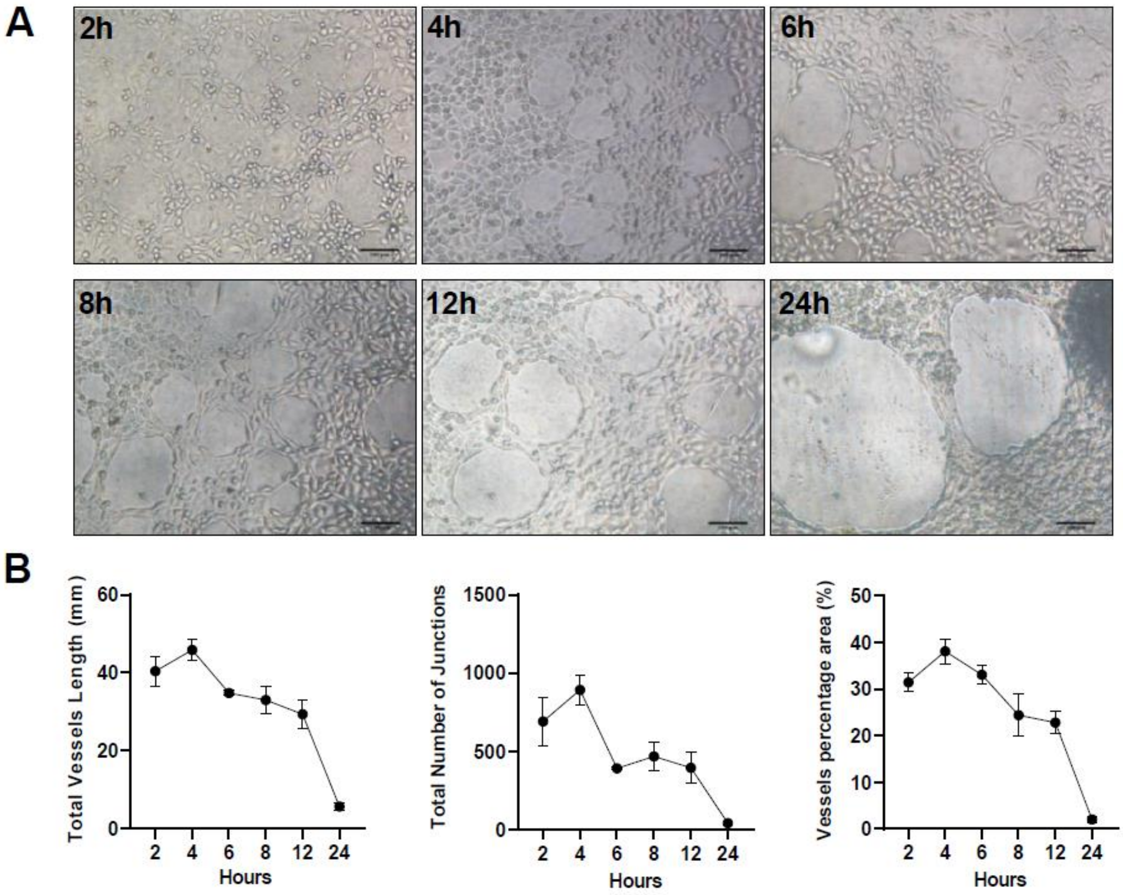

Figure S5. Pilot time-screening experiments for tubule formation test. (A) Representative images of tubule formation at 2, 4, 6, 8, 12, 24 hours after HUVECs seeding. (B) Quantification of tubule formation in three independent experiments.

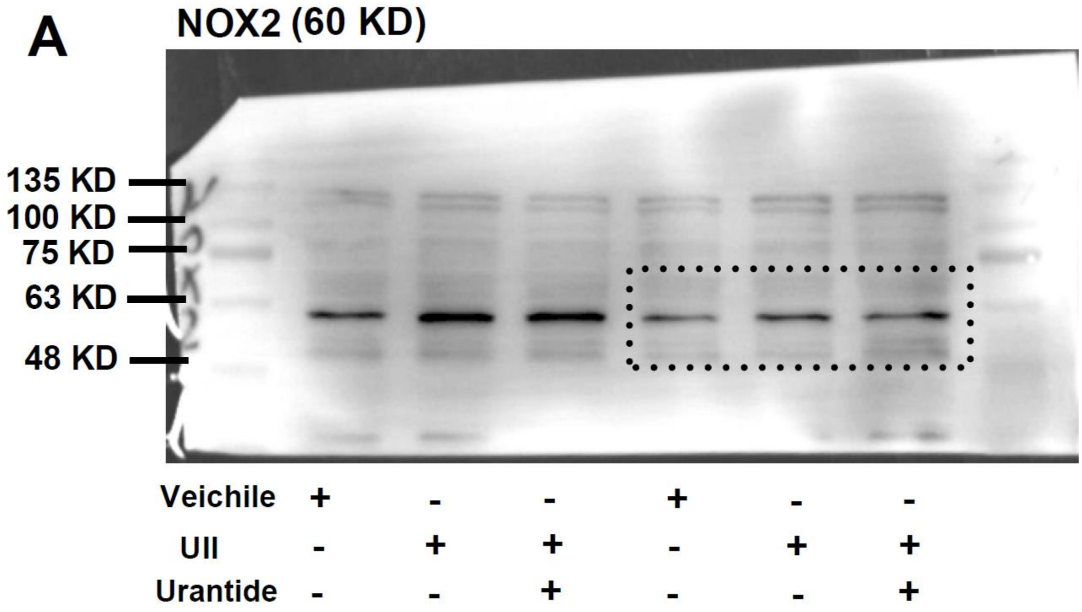

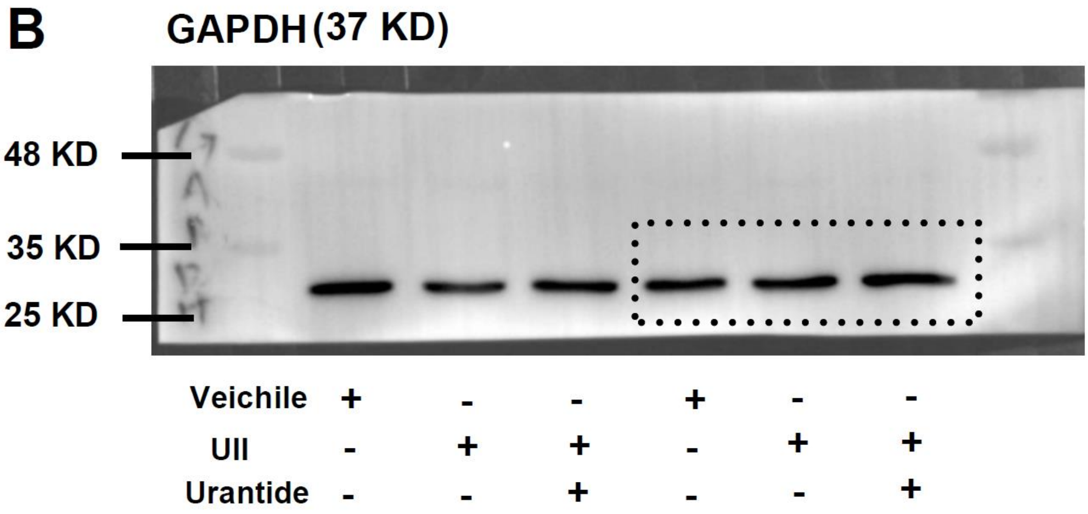

Figure S6. Original uncropped Western blots for Figure. 6A.

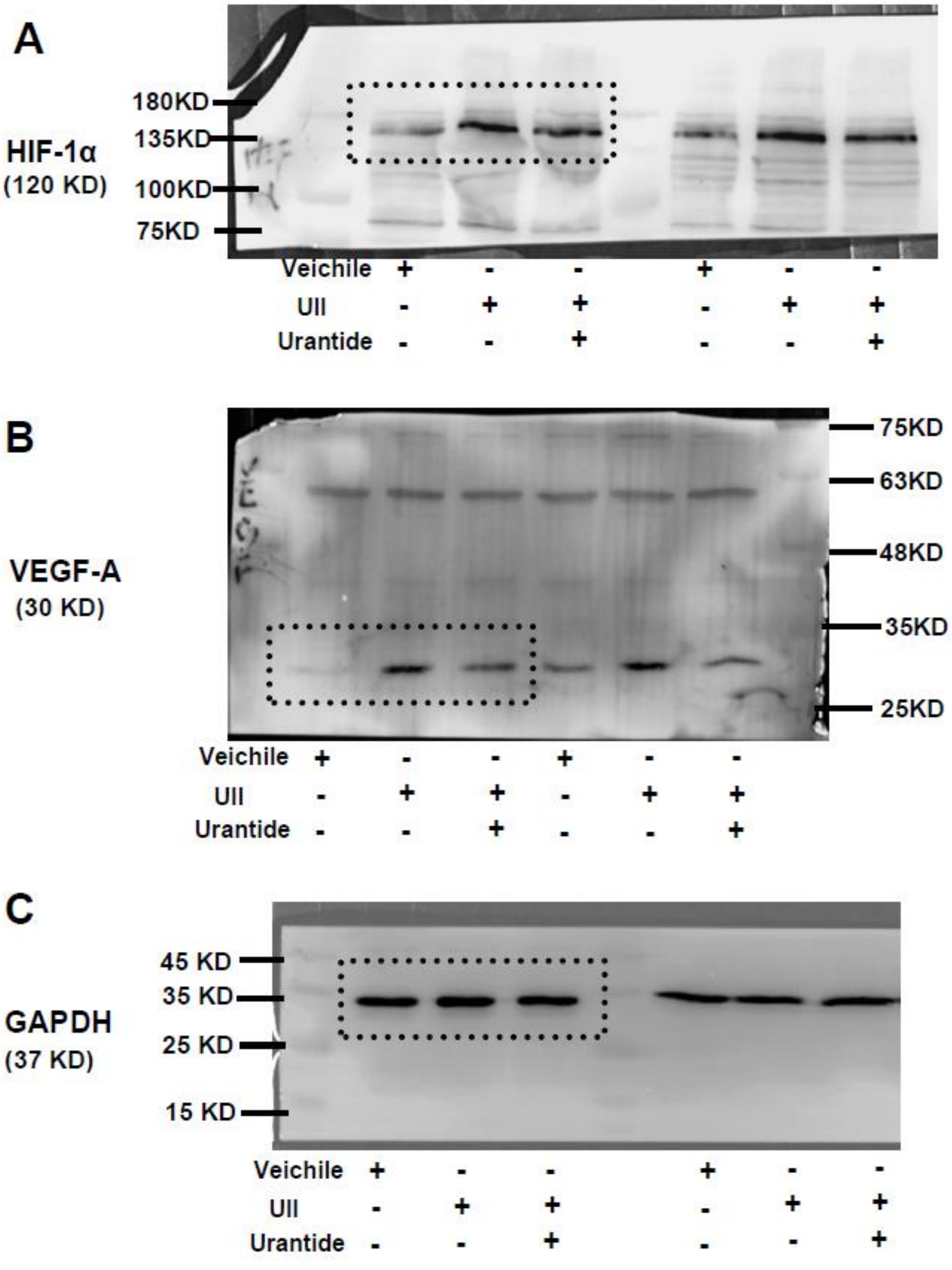

Figure S7. Original uncropped Western blots for Figure. 6D.
